# The relationship between night work and breast cancer

**DOI:** 10.1186/s40557-018-0221-4

**Published:** 2018-02-06

**Authors:** Hye-Eun Lee, Jongin Lee, Tae-Won Jang, In-Ah Kim, Jungsun Park, Jaechul Song

**Affiliations:** 1 Department of Occupational and Environmental Medicine, Kyung Hee University Hospital, Dongdaemun-gu Kyunghee-daero 23, Seoul, Republic of Korea; 2 Department of Occupational and Environmental Medicine, College of Medicine, The Catholic University of Korea, Seoul, Republic of Korea; 3 Department of Occupational and Environmental Medicine, Hanyang University College of Medicine, Seoul, Republic of Korea; 4 Department of Occupational Health, Catholic University of Daegu, Gyonsan-si, Republic of Korea

**Keywords:** Breast neoplasm, Night work, Carcinogens

## Abstract

**Background:**

Since the International Agency for Research on Cancer classified shift work that involves circadian disruption as “probably carcinogenic to humans,” there has been growing concern on the relationship between night work and breast cancer. In Korea, about 10–15% of workers are engaged in night-shift work, and breast cancer is one of the most common cancers in women. The purpose of this study was to review epidemiologic evidence on the relationship between night work and breast cancer.

**Methods:**

We reviewed 21 original articles and 5 meta analyses on relationship between nightwork and breast cancer, and investigated the compensation criteria of Denmark.

**Results:**

The association between breast cancer and night work has been reported by numerous epidemiologic studies, including cohort studies, case-control studies, and meta-analysis. However, a dose-response relationship has not clearly emerged among workers exposed to less than 20 years of night work.

**Conclusion:**

Although there are some limitations to the epidemiological studies so far, further consideration of breast cancer cases in patients with high exposure to night work is needed to assess breast cancer as a work-related disease.

## Backgrounds

Shift work that involves circadian disruption has been classified as “probably carcinogenic to humans” (group 2A) by the International Agency for Research on Cancer (IARC) [1, 2]. This decision, made in 2007, was based on sufficient evidence from experimental studies, but limited evidence from epidemiological studies. Since then, attention to breast cancer in night workers has been growing. In addition, in Korea, the number of cases of patients with breast cancer who have been exposed to night work and who thereby apply for compensation for breast cancer as an occupational disease is increasing. The International Labor Organization reports that more than two and a half billion people are engaged in shift work that involves night work [2].

Considering the higher incidence of breast cancer and prevalence of night work, it has become a major priority to examine evidence of night work-related breast cancer.

The aim of this study is to review epidemiologic evidence on the relationship between night work and breast cancer.

**Table 1:** Cohort studies of night work and breast cancer

Authors, (years) Country	Study description	Exposure assessment	Exposure categories	RR or HR
Schernhammer et al. (2001) [13] USA	Prospective cohort study of 121,701 registered nurses, with follow-up 1988–1998	Self-reported lifetime years on rotating night shifts, with rotating night shifts defined as “at least three nights per month, in addition to evenings and afternoons in that month”	Never 1–15 15–29 ≥ 30 *P* for trend	1.0 (ref) 1.08 (0.99–1.18) 1.08 (0.90–1.30) 1.36 (1.0–1.78) 0.02
Schernhammer et al. (2006) [14] USA	Prospective cohort study of 116,087 registered nurses, with follow-up 1989–2001	Self-reported lifetime years on rotating night shifts, with rotating night shifts defined as “at least three nights per month, in addition to evenings and afternoons in that month”	Never 1–9 10–19 20+ *P* for trend	1.0 0.98 (0.87–1.10) 0.91 (0.72–1.16) 1.79 (1.06–3.01) 0.65
Schwartzbaum et al. (2007) [15] Sweden	Register-based retrospective cohort study of 1,148,661 female workers, with follow-up 1971–1989	Usual occupation and work hours (three-shift schedules and others) to define occupations with a large proportion of workers with night work; from in-person interviews in annual surveys of living conditions (1977–1981)	Shift work in 1970 Shift work in both 1960 and 1970	0.94 (0.74–1.18) 0.97 (0.67–1.40)
Pronk et al. (2010) [18] China	Shanghai Women’s Health Study: a population-based prospective cohort study	Job exposure matrix of three categories with increasing scores for night-shift work: 0 = no night-shift work; 1 = incidental night-shift work; 2 = jobs likely to involve the night shift, 3 = jobs that probably involved all-night shifts	Never 1–5 years 6–17 years 18 years and more	1.0 (ref) 0.9 (0.6–1.3) 0.9 (0.6–1.4) 0.8 (0.5–1.2)
Knutsson et al. (2012) [16] Sweden	WOLF (Work, Lipids, and Fibrinogen) occupational cohort study that included subjects who were employed in different public and private companies (*N* = 4036), with average follow-up time of 12.4 years	Questionnaire: “Do you work shifts?” and “How many hours do you normally work per week, including overtime, and how are these hours distributed on average?”	Day Shifts without nights Shifts with nights	1.0 (ref) 1.23 (0.70–2.17) 2.02 (1.03–3.95)
Koppes et al. (2014) [19] Netherlands	14 Dutch Labor Force Surveys (1996–2009) Record linkage with national registers on hospital admission	Current exposure to night work was assessed with the question: “Do you work at nights, meaning between midnight and 6 am?”	No night work Occasional Regular	1.0 (ref) 1.04 (0.85–1.27) 0.87 (0.72–1.05)
Åkersted et al. (2015) [17] Sweden	Cohort study of 13,656 women from the Swedish Twin Registry, with 3404 exposed to night work; follow-up time of 12 years	Questionnaire: “For how many years have you had working hours that meant that you worked nights at least now and then?”	Follow-up to 60 years	
			No night work	1.0 (ref)
			1–5 years	0.93 (0.66 to 1.31)
			6–10 years	0.79 (0.45 to 1.38)
			11–20 years	0.80 (0.45 to 1.42)
			21–45 years	1.77 (1.03 to 3.04)

*RR* Relative risk, *HR* Hazard ratio

**Table 2:** Case-control studies of night work and breast cancer

Authors, (years) Country	Study description	Exposure Assessment	Exposure categories	Odds ratio
Tynes et al. (1996) [27] Norway	Nested case-control study of a cohort of 2619 female radio and telegraph operators enrolled 1920–1980, with follow-up 1961–1991; 50 cases and 4–7 matched (year of birth) controls	Collected detailed job histories from Norwegian seamen registry; “Work at night with exposure to artificial light?” From cases and controls, detailed information on job histories on ships, as well as shift work and travel through time zones was collected	*Aged* < *50*	
			None	1.0 (ref)
			< 3.1 years.	0.3 (0.1–1.2)
			> 3.1 years	0.9 (0.3–2.9)
			*P* for trend	0.97
			*Aged 50+*	
			None	1.0 (ref)
			< 3.1 years	3.2 (0.6–17.3)
			> 3.1 years	4.3 (0.7–26.0)
			*P* for trend	0.13
Hansen (2001) [20] Denmark	Nested case-control study with follow-up 1964–1999; 7565 cases and 1:1 matched controls (year of birth and sex)	Individual employment histories were obtained from files of national pension funds	All night work combined in trades with > 60% night work	1.5 (1.3–1.7)
			Employed > 6 years	1.7 (1.3–1.7)
			Nurses	1.3 (1.1–1.4)
Davis et al. (2001) [21] USA	Cancer register-based case–control study; 813 cases (1992–1995) and 793 matched (5-year age groups) controls identified by random-digit dialing	Information on sleeping habits, light exposure, lifetime occupational history obtained from in-person interviews; night workers defined if ≥1 graveyard shift/wk. (8 h) in 10 years before diagnosis of cancer	*Years worked* ≥ *3 nights/wk.*	
			None	1.0 (ref)
			< 1	1.2 (0.6–2.3)
			1–3	1.4 (0.7–2.8)
			3–4.6	0.6 (0.3–1.5)
			4.7+	2.3 (1.2–4.2)
			*P* for trend	0.01
Lie et al. (2006) [22] Norway	Nested case-control study of the cohort of 44,835 Norwegian nurses; 537 cases (1960–1982) and 1:4 matched (year of birth) controls	Total work history reconstructed from occupational information for nurses from the registry censuses of the Norwegian Board of Health in 1960, 1970, and 1980	*Years night work*	
			0	1.0 (ref)
			1–14	0.95 (0.67–1.33)
			15–29	1.29 (0.82–2.02)
			30+	2.21 (1.10–4.45)
			*P* for trend	0.01
O’Leary et al. (2006) [29] USA	Case-control study of 576 cases (1996–1997) and 585 1:1 matched (age in 5-year age groups) population-based controls	Occupational history since age 16, and residential light-at-night exposures (e.g., sleep hours, frequency of turning on lights during night, length of time light was on) from in-person interviews	Any evening or overnight shift work	1.04 (0.79–1.38)
			Any evening shift work only	1.21 (0.90–1.64)
			Any overnight shift work only	0.55 (0.32–0.94)
Pesch et al. (2010) [30] Germany	GENICA: a population-based case-control study conducted among women from the Greater Region of Bonn, Germany	Night work was defined as working full-time between 24.00–05.00 h	Never	1.0 (ref)
			1–4 years	0.64 (0.34–1.24)
			5–9 years	0.93 (0.41–2.15)
			10–19 years	0.91 (0.38–2.18)
			20 years and more	2.49 (0.87–7.18)
Lie et al. (2011) [26] Norway	Nested case-control study within a cohort of 49,402 female nurses; 699 cases and 895 controls	“Night work” includes working periods in both rotating and permanent night schedules, and includes the work of permanent night workers	Never night work	1.0 (ref)
			1–11 years	1.2 (0.9–1.5)
			12 years	1.3 (0.9–1.8)
			*P* for trend	0.17
Hansen et al. (2012) [23] Denmark	Nested case-control study within a nationwide cohort of Danish nurses (*N* = 91,140), including detailed information on lifetime shift-work and potential confounders	Information on shift work obtained from interviews; Day work defined as working from 6 or 7 to 15 or 16, evening work from 15 or 16 to 23 or 24, and night work from 23 or 24 to 7 or 8	Day-evening only	1.0 (ref)
			1–4 years of night work	1.5 (0.99–2.5)
			5–9	2.3 (1.4–3.5)
			10–19	1.9 (1.1–2.8)
			≥ 20	2.1 (1.3–3.2)
Hansen et al. (2012) [24] Denmark	Nationwide case-control study nested within a cohort of 18,551 female military employees born in 1929–1968	Information on shift work, sun exposure habits, diurnal preferences, and other potential confounders	Never	1.0 (ref)
			1–5.9 years	0.9 (0.4–1.7)
			6–14.9 years	1.7 (0.9–3.2)
			15 years and more	2.1 (1.0–4.5)
			*P* for trend	0.06
Fritschi et al. (2013) [28] Australia	Case control study of cases from the population-based Western Australian (WA) Cancer Registry, with 1205 incident cases and 1789 frequency age-matched controls	Information on shift work obtained from telephone interviews, with levels of night work being high: job involved > 4 nights forward rotation or > 6 nights backward rotation, medium: 3–4 nights forward or 4–6 nights backward rotation, or low: 3 nights backward rotation	No rotation Ever < 10 years 10–20 years > 20 years	1.0 (ref) 1.16 (0.97–1.38) 1.25 (1.00–1.56) 1.09 (0.79–1.50) 1.02 (0.71–1.45)
Grundy et al. (2013) [25] Canada	A case-control study from 2005 to 2010	Case definition: where ≥50% of time was reported to have been spent on evening and/or night shifts, capturing both rotating and permanent night shift schedules	No shifts	1.0 (ref)
			0–14 years	0.95 (0.79–1.16)
			15–29 years	0.93 (0.67–1.30)
			> 30 years	2.21 (1.14–4.31)
Menegaux et al. (2013) [31] France	Population-based case-control study with 1232 cases of breast cancer and 1317 population controls	Information on shift work obtained from in-person interviews; Overnight: night shift of 6 consecutive work hours or more spanning the time period 11 pm–5 am	Never	1.0 (ref)
			< 4.5 years	1.27 (0.83–1.94)
			4.5 years and more	1.40 (0.96–2.04)
Li et al. (2015) [32] China	An extension of a series of case-cohort studies of textile industry exposures to dusts, chemicals, and other physical agents in relation to risks of various cancers	Night work was defined as continuous work between 24:00 and 05:00	Day workers	1.0 (ref)
			3 times/month	1.4 (0.8–2.6)
			1–14 years	1.2 (0.9–1.6)
			15–29 years	1.2 (0.9–1.7)
			> 30 years	0.8 (0.5–1.4)
Papantoniou et al. (2015) [33] Spain	Population-based case-control study with 1708 breast cancer cases and 1778 population controls from 10 Spanish regions	Lifetime occupational history was assessed by face-to-face interviews	Never night work	1 (ref)
			Ever night work	1.18 (0.97, 1.43)
			Permanent	1.19 (0.89, 1.60)
			Rotating	1.17 (0.91, 1.51)

**Table 3:** Meta-analysis of night work and breast cancer

Authors (years)	Overall OR or RR (95% CI)	Night work exposure category
Megdal et al. (2005) [34]	1.51 (1.36–1.68)	Ever
Jia et al. (2013) [35]	1.20 (1.08–1.33)	Ever
	1.15 (1.03–1.29)	≥ 15 years
Kamdar et al. (2013) [36]	1.21 (1.00–1.47)	Ever
	1.04 (0.92–1.18)	≥ 8 years
Wang et al. (2013) [37]	1.19 (1.05–1.35)	Ever
	1.03 (1.01–1.05)	Every 5 years of exposure increased
Ijaz et al. (2013) [38]	1.05 (1.01–1.10)	Every 5 years of exposure increased
Travis et al. (2016) [39]	0.99 (0.95–1.03)	Ever
	1.01 (0.93–1.10)	≥ 20 years
	1.00 (0.87–1.14)	≥ 30 years

*OR* Odds ratio, *RR* Relative risk

## Methods

To review scientific evidence for a relationship between night work and breast cancer, we searched databases, including Ovid-MEDLINE, EMBASE, and PubMed, without date or language restrictions. We looked over the references from included studies and existing systematic reviews. We reviewed original articles including 7 cohort studies, 14 case-control studies and 6 meta analyses, and additionally, we attempted to obtain more information on each of the studies from systematic reviews, including the IARC monograph [2] and meta-analyses.

To understand the epidemiology of breast cancer, the status of night work in Korea, and compensation criteria for breast cancer in night workers in other countries, we reviewed government reports and various grey articles in addition to scientific articles.

## Results and discussion

### Incidence and risk factors for breast cancer

According to Korea National Cancer Incidence Database, the crude incidence rate of breast cancer was 68.2 per 100,000 patients, which was the second most common cancer in women following thyroid cancer in 2013. The age standardized incidence rate of breast cancer per 100,000 patients increased from 12.5 in 1999 to 26.2 in 2013. The annual percentage change was calculated as 5.6%, which was also the second most rapid increase in cancer following thyroid cancer.

Risk factors of breast cancer include exposure to endogenous and exogenous female sex hormones, lifestyle-related factors such as drinking alcohol or having low levels of physical activity, and hereditary factors [3]. These established risk factors, however, contribute to only about 50% of the cases of breast cancer [4]. As a result, efforts have been made to explore other risk factors for breast cancer, including occupational or environmental factors.

### Definition of night work

The meaning of the term “period of night work” varies from country to country. For example, definitions of night time range from 00:00 to 05:00 in France and the UK, between 20:00 and 06:00 in Belgium, and between 20:00 and 07:00 in Portugal [2]. According to the Labor Standard Act, night work in Korea is defined as work between 22:00 and 6:00. Generally, these definitions have been established for the regulation of work hours and compensation for work performed at non-standard working hours.

With the increasing amount of research on the health effects of night work, there is a need for a consistent definition of night work for purposes of epidemiologic investigation regarding the biological effects of disruption to circadian rhythms in night workers. Accordingly, the IARC assembled a workshop to discuss how “shift work” should be assessed. In this workshop, the IARC proposed which domains of non-day shifts and shift schedules should be examined, including (1) shift systems, (2) years on non-day shift schedules, and (3) shift intensity (i.e., time off between work days). The suggested definition of night work is “at least three hours of work between 00:00 and 05:00” [5].

### Exposure to night work in Korea

Shift work that involves night work is essential for some public services, such as the provision of transportation, gas, electricity, and health care. Shift work is inevitable for the technological needs of some industries, including power plants, oil refineries, and the steel industry. In some cases, shift work is utilized to achieve higher productivity by operating machines for 24 h, typically in manufacturing industries.

Several surveys include the investigation of night work in Korea. Night workers are estimated to comprise about 1.27 million workers (11.2% of total workers), 1.97 million workers (14.5%), and 1.34 million workers (10.2%) in the Survey Report on Labor Conditions by Employment Type (2010), the Korea National Health and Nutrition Examination Survey (2007–2009), and the Korean Labor and Income Panel Study (2008), respectively [6]. In these surveys, manufacturing industries (employing approximately 456,000 night workers), transportation (200,000 workers), and human health and social care activities (90,000–130,000 workers) are the main industries with significant proportions of night workers. In the Korean Working Condition Survey, 13.2% of the working population is recognized as night workers, with night work defined as at least two hours of work between 22:00 and 05:00. In this survey, among employed workers, 17.4% of male workers and 7.5% of female workers are night workers [7]. According to the fourth European working condition survey, the prevalence of shift work, including night work, was approximately 20% of employees in European countries in 2005 [2]. The prevalence of night work in Korea is lower than in European countries, with one potential reason for this being the extremely long working hours of Korean workers in general. Moreover, in 2012, the most common shift system in Korea was a two-team, 12-h shift system (prevalent in 60% of businesses with shift systems), followed by a two-team, 24-h shift system (in 14.6% of businesses) [8]. This means that the majority of night-shift workers in Korea are exposed to a higher intensity of night work or, in other words, a greater amount of night-shift work per month and year.

### Biological plausibility

Numerous experimental studies support the notion that melatonin produces a potent circadian anti-cancer signal to cancer cells and protects normal cells from initiation [9]. In many experimental animal studies, an accelerated growth of mammary tumors has been found in response to exposure to constant light at night and/or in animals with a status of surgical pinealectomy [2]. During the natural darkness of night, pineal glands produce high levels of melatonin. However, reduced melatonin production has been reported among night-shift workers [10–12]. Consequently, the suppression of melatonin secretion by exposure to light during nighttime hours is surmised to be the main biological mechanism in the relationship between breast cancer and night work. In conclusion, the biological mechanism of the relationship between breast cancer and night work is explained with sufficient evidence from animal studies and experimental data.

### Scientific evidence for the relationship between night work and breast cancer

The association between breast cancer and night work is reported in numerous epidemiologic studies, although the magnitude of association is not substantial. A dose-response relationship is not clear among workers exposed to less than 20 years of night work. So far, the most significant limitations in epidemiological studies are different definitions of night work and varied exposure assessment across studies. Consistency in studies according to the objective assessment of exposure to night work is required.

#### Cohort studies (Table 1)

An IARC evaluation from 2007 includes three cohort studies [13–15]. Two of the studies show an increased relative risk for breast cancer in the range of approximately 1.4–1.8 among female workers with more than 20–30 years of night work [13, 14]. Since this IARC evaluation, four more cohort studies have been published. Generally, recent studies tend to obtain more detailed information on night work and possible confounders in comparison to older studies. Statistically significant risks are found in two of the four recent studies, with a range of approximately 1.8–2.0, in comparative analysis of groups exposed to extreme levels of night work [16, 17]. The other two studies show no overall effects of night-shift work [18, 19]. On the whole, four of seven cohort studies show increased risks of night work on breast cancer in sample populations of female night workers. Two studies with nurses’ cohort showed significantly increased risk [13, 14]. The other cohort studies were built with working population with various jobs [15–19].

#### Case-control studies (Table 2)

There are more case-control studies, including nested case-control studies, regarding the effects of night work on breast cancer than there are cohort studies on the topic. Including five studies evaluated by the IARC in 2007, six of fourteen case-control studies show significantly increased risks of cancer in high-exposure groups for night work [20–25]. Half of the studies were population based case-control studies, three studies were nested case-control studies in nurses’ cohorts [22, 23, 26]. Two studies were nested case-control studies in cohorts of the other occupations including radio and telegraph operators and military employees [24, 27].

In a study by Fritschi et al., only a lower-exposure group shows a significantly increased risk of cancer due to night work [28]. The other studies report non-significantly increased risks with odds ratios (ORs) not higher than 1.50, except for in two studies [26, 27, 29–33].

#### Meta-analysis (Table 3)

The first meta-analysis reported in 2005 includes six studies, and finds an increased risk for breast cancer among night workers (relative risk [RR], 1.51; 95% confidence interval: 1.36–1.68) [34]. After the IARC evaluation, four meta-analysis studies on breast cancer and night work were published in 2013. According to these studies, breast cancer risks due to night work are significantly increased, and the pooled RR ranges from 1.19 to 1.21 [35–37]. In two studies, the risk for breast cancer is estimated to increase 3–5% with every five years of exposure to night work [37, 38]. A recent meta-analysis study didn’t show effect of night work on breast cancer [39].

### Criteria for compensation

In Denmark between 2007 and 2011, the work-relatedness of breast cancer with night work was recognized in 110 cases, and the patients were therefore eligible for compensation by the Danish National Board of Occupational Injuries [40]. The patients in these cases had occupational histories of more than 20 years of shift work that involved night work more than once a week. However, the Danish National Board of Industrial Injuries and the Occupational Diseases Committee reported a change in practice for claims regarding breast cancer and night-shift work in 2013. Following the new criteria, cases with at least 25 years of regular night-shift work may be submitted to the Occupational Diseases Committee for consideration of provision of worker compensation [41]. Except in Denmark, it is difficult to find a country in which breast cancer-related night work is officially compensated.

## Conclusions

Breast cancer in patients with high exposure to night work should be understood to be an occupational disease, and patients should be eligible for workers’ compensation, as in Denmark. In Korea, general working hours are longer and night shifts for shift workers are more frequent than in European countries. Therefore, various factors, such as total working hours, the frequency of night work, work schedules (including rotating schedules and rest periods after night shifts), and co-exposure to other occupational carcinogens additional to years of employment in non-day shift work must be considered. Furthermore, restrictions on the frequency of night shifts or exposure periods to night work might be considered in order to reduce the risk of breast cancer among night workers in Korea.

## Abbreviations

IARC: The International Agency for Research on Cancer
